# Electronic Structure and Surface Chemistry of BaZrS_3_ Perovskite Powder and Sputtered Thin Film

**DOI:** 10.1021/acsami.4c06758

**Published:** 2024-07-22

**Authors:** Stefania Riva, Soham Mukherjee, Sergei M. Butorin, Corrado Comparotto, Garima Aggarwal, Evelyn Johannesson, Mahmoud Abdel-Hafiez, Jonathan Scragg, Håkan Rensmo

**Affiliations:** † Division of X-ray Photon Science, Department of Physics and Astronomy, Uppsala University ,Box 516, Uppsala SE-75120, Sweden; ‡ Division of Solar Cell Technology, Department of Materials Science and Engineering, Uppsala University, Uppsala 75237, Sweden; § Department of Applied Physics and Astronomy, University of Sharjah, P.O. Box 27272, Sharjah ,United Arab Emirates; ∥ Wallenberg Initiative Materials Science for Sustainability (WISE), Department of Physics and Astronomy, Uppsala University, Uppsala SE-75120, Sweden

**Keywords:** BaZrS_3_, chalcogenide perovskites, XPS, HAXPES, electronic structure, DFT

## Abstract

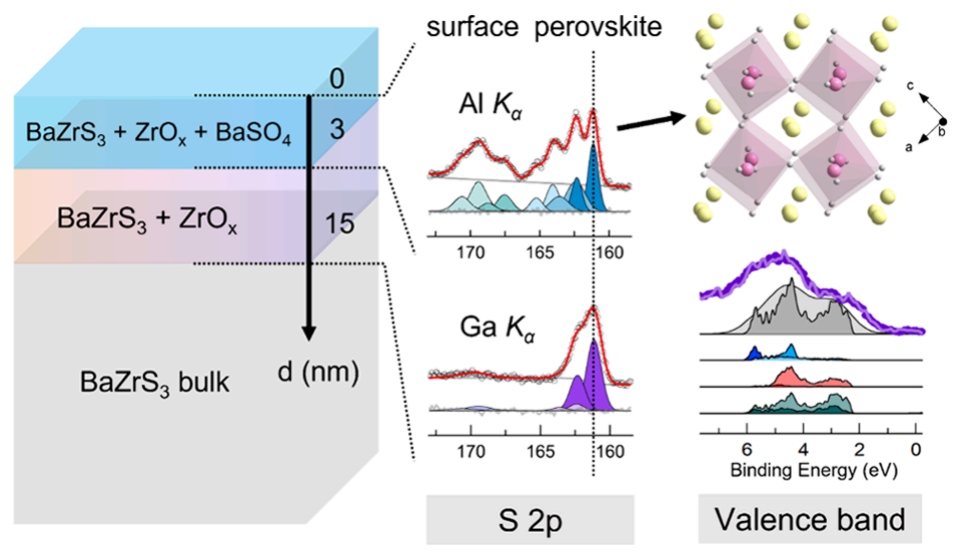

Chalcogenide perovskites exhibit optoelectronic properties that position them as potential materials in the field of photovoltaics. We report a detailed investigation into the electronic structure and chemical properties of polycrystalline BaZrS_3_ perovskite powder by X-ray photoelectron spectroscopy, complemented by an analysis of its long- and short-range geometric structures using X-ray diffraction and X-ray absorption spectroscopy. The results obtained for the powdered BaZrS_3_ are compared to similar measurements on a sputtered polycrystalline BaZrS_3_ thin film prepared through rapid thermal processing. While bulk characterization confirms the good quality of the powder, depth-profiling achieved by photoelectron spectroscopy utilizing Al *K*_α_ (1.487 keV) and Ga *K*_α_ (9.25 keV) radiations shows that, regardless of the fabrication method, the oxidation effects extend beyond 10 nm from the sample surface, with zirconium oxides specifically distributing deeper than the oxidized sulfur species. A hard X-ray photoelectron spectroscopy study on the powder and thin film detects signals with minimal contamination contributions and allows for the determination of the valence band maximum position with respect to the Fermi level. Based on these measurements, we establish a correlation between the experimental valence band spectra and the theoretical density of states derived from density functional theory calculations, thereby discerning the orbital constituents involved. Our analysis provides an improved understanding of the electronic structure of BaZrS_3_ developed through different synthesis protocols by linking it to material geometry, surface chemistry, and the nature of doping. This methodology can thus be adapted for describing electronic structures of chalcogenide perovskite semiconductors in general, a knowledge that is significant for interface engineering and, consequently, for device integration.

## Introduction

Chalcogenide perovskites (chemical formula ABQ_3_) are a notable perovskite subgroup where divalent cations Ba, Sr, Ca, and Eu occupy the A-site; tetravalent Zr or Hf occupy the B site, while divalent anion Q may be either S and/or Se. Among all the combinations, BaZrS_3_ has emerged as one of the most promising compounds in photovoltaic research due to its phase stability against air, moisture and high pressure,^1−3^ abundance of constituent elements in the Earth’s crust and safety considerations.^4^ Numerical simulations of a solar cell device with BaZrS_3_ in the form of FTO/ZrS_2_/BaZrS_3_/SnS/Aupredict an efficiency as high as 28.08%.^5^ At the current stage, the applicability of BaZrS_3_ for photovoltaic technologies has been studied with photodetectors, which have shown stability over time but exhibit a high dark current, probably related to a high density of defects.^6,7^ Furthermore, recently Dallas et al. have processed a first operational solar cell device with a redox electrolyte, showing a mean efficiency of 0.11% and a mean fill factor of 61%.^8^

Crystalline BaZrS_3_ assumes a distorted orthorhombic structure, where the Ba^2+^ cations are embedded in cuboctahedral voids formed by corner-shared octahedral units of Zr–S_6_, with an average cooperative tilting angle of ∼10.2°.^9^ In terms of the optical properties, the BaZrS_3_ band gap is between 1.7 and 1.9 eV, and its absorption coefficient is >10^5^ cm^–1^,^3,6^ making it suitable for photovoltaic applications in a tandem structure.^10^ The theoretical study by Nishigaki et al.^10^ indicates that the high absorption coefficient arises mainly from the electronic transition from the highly populated state of S 3p to the empty Zr 4d orbitals in the conduction band. Density of states (DOS) calculations on BaZrS_3_ in the GdFeO_3_-type perovskite structure reveal the valence band to be composed mainly of high-density states of S 3p and Zr 4d, while the main conduction band states are S 3p, Ba 5d and Zr 4d orbitals.^10−12^

The scientific community has devoted considerable efforts toward synthesizing stoichiometric BaZrS_3_ thin films at low temperatures using various fabrication methods, such as sputtering,^13,14^ molecular beam epitaxy,^15^ pulsed laser deposition^6^ and bulk solid-state methods.^16^ X-ray diffraction (XRD) confirms the successful formation of BaZrS_3_, while some observations indicate partial replacement of S with O atoms,^17^ and/or the possible formation of ZrO_2_.^18^ Although stoichiometric bulk samples demonstrate resistance to bulk oxidation even at high temperature,^1^ the oxyphilic nature of the constituent elements, particularly Zr, appears to result in some surface oxidation. This can be observed, for example, through TEM analysis, where oxygen and an off-stoichiometric composition are detected in the topmost few nanometers of BaZrS_3_ thin films.^15,18^ Whether the oxidation occurs during synthesis itself or upon subsequent air exposure can be difficult to discern, but in either case, it modifies the surface chemistry. A thorough depth-profiling of chemical speciation and structural properties, along with the impact of such varied surface chemistry on the electronic structure of BaZrS_3_, is not properly understood. Investigation of the BaZrS_3_ surface is a key to understand the material behavior in the context of device fabrication, as the electronic structure and interfacial energetics can strongly influence the extraction of charge carriers.

X-ray photoelectron spectroscopy (XPS) is a nondestructive surface sensitive technique with a maximum probing depth of ∼15 nm, which can help determine the electronic structure, chemical composition and electron dynamics occurring at surfaces and buried interfaces. By varying the photon energy, it is possible to distinguish chemical species at different depths within the material. Furthermore, XPS allows for experimentally probing the DOS at the occupied valence band which can be mapped onto the theoretically calculated band structure to identify the element-specific atomic orbitals contributing to the sample properties. By fitting the valence band edge, one can determine the position of the valence band maximum (VBM) with respect to the Fermi level, important for evaluating the materials’ candidacy for forming junctions, and is crucial for constructing energy levels diagrams when BaZrS_3_ is combined with other materials in devices.^12,19^

In this work, polycrystalline BaZrS_3_ samples were investigated in powder and thin film forms. XPS measurements for the powder were acquired at two photon energies: soft X-rays (Al *K*_α_, 1.487 keV) and hard X-rays (Ga *K*_α_, 9.25 keV, also known as Hard X-ray Photoelectron Spectroscopy or HAXPES), allowing us to perform depth-profiling of the sample surface. Utilizing the higher depth sensitivity of the Ga *K*_α_ source, a comparison of the materials chemistry of the powder and the thin film is reported. The nature of doping in both semiconductors was determined by evaluating the VBM position relative to the Fermi level within the material band gap. The experimentally determined valence bands of the samples were compared to the valence band spectrum obtained from DFT calculations, thereby resolving the different orbital components of Ba, Zr and S. In addition, the relative contributions of different orbitals to the valence band spectrum, their relation to the distorted orthorhombic geometry, and relevance to the nature of doping in semiconducting BaZrS_3_ are discussed. The present paper aims to serve as a reference for future spectroscopy studies on the interface and electronic structure of BaZrS_3_, as well as other chalcogenide perovskites, which may help the interfacial control for the design of devices.

## Methods/Experimental Section

### Synthesis of Powder and Characterization

All preparation steps of weighing, mixing, grinding, and storage were conducted inside an argon-filled glovebox, with oxygen (O_2_) and water (H_2_O) levels maintained at less than 0.1 ppm. The BaZrS_3_ powder was synthesized within an evacuated silica-glass ampule^20^ (Figure S1a in the Supporting Information) using pure elements: Ba (0.5 mm granules, Merck, 99.9%), Zr powder (99.5%), and S powder (Labtex, 99.5%). The elements were precisely weighed in an atomic ratio of 1:1:3 and carefully sealed in a quartz ampule. Subsequently, the ampule was evacuated and sealed before being placed in a horizontal tube furnace. The temperature was gradually increased to 750 °C over several weeks. The outcome of this process was a mixture of barium and zirconium sulfides, with no traces of elemental sulfur detected. To obtain the final product of BaZrS_3_, the substance was then ground in an inert atmosphere (Ar) and transferred to another quartz glass ampule. Following this, it went through annealing for several weeks at 750 °C, facilitating further structural development and refinement. This multistep synthesis process aimed to ensure the purity and stability of the BaZrS_3_ powder. The structural properties of the obtained BaZrS_3_ powder sample were analyzed by X-ray diffraction (model: Bruker D8) with Cu *K*_α_ radiation (1.4506 Å), allowing the determination of the lattice constants of the unit cell.

### Synthesis of Thin Film and Characterization

The thin film was prepared on a silicon substrate via sputtering from BaS and Zr targets in an atmosphere of H_2_S and Ar, with a subsequent annealing process at 900 °C in N_2_, as described in our previous report.^13^ The sample was identified to form at this optimum temperature for the given synthetic process, based on the analysis of photoluminescence, XRD, XAS and XPS data for a series of samples.^13,21^

### X-ray Absorption Spectroscopy (XAS)

The Zr *K*-edge for BaZrS_3_ powder was measured by XAS at the Deutsches Elektronen-Synchrotron DESY (Hamburg, Germany) at the beamline P64,^22^ Hamburg, using high photon flux (∼10^12^ photons/s) on the sample and high resolution (Δ*E*/*E* ≈ 10^–4^). The incoming photon beam was monochromatized through a Si (111) double crystal monochromator. N_2_ gas was used in the ion chambers to absorb ∼5% of the flux, and an identical setup was used for the transmission and the reference channels, with the latter one hosting Zr metal foil to account for any energy offsets. The powder was mixed with boron nitride and calculated for an edge jump of ∼1.5, pressed into pellets, placed on Kapton tape, and mounted at 45° to the incoming X-ray beam, allowing simultaneous measurements in transmission and fluorescence modes. A PIPS detector was used to collect the fluorescence data. The XAS data was collected to achieve good statistics up to ∼1000 eV beyond the Zr *K*-edge position, which translates to wavenumbers as high as ∼16 Å^–1^. The corresponding Zr *K*-XAS measurements on the sputtered thin films of BaZrS_3_ were performed at the BALDER beamline, as reported previously.^21^ The XAS data sets were processed and analyzed using the Athena-Artemis software suite, a user interface for FEFF and IFEFFIT.^23^ The subtracted background was computed using AUTOBK algorithm implemented in the software.^24^ The uncertainties associated with extracted EXAFS parameters were calculated using a standard Levenberg–Marquardt, nonlinear minimization of the statistical χ^2^ parameter, built within the FEFF program. Detailed information regarding the extracted EXAFS parameters and their corresponding error margins, can be found in the Supporting Information.

### X-ray Photoelectron Spectroscopy

XPS measurements were performed at the Kai-Siegbahn laboratory at Uppsala University. The laboratory is equipped with two monochromatic photon sources, Al *K*_α_ (1.487 keV) and Ga *K*_α_ (9.25 keV), and a Scientia EW4000 spectrometer. For the characterization of the BaZrS_3_ powder, the different sources were used to get a depth-profile view. Due to the poor conductivity of the powder sample, it was spread uniformly and pressed on a soft indium foil that was mounted on a metal sample holder. To ensure charge neutralization, the low (Al *K*_α_) and high (Ga *K*_α_) photon energy measurements on the powder were performed with the aid of a flood gun with an energy in the range of about 0.5 and 3.65 V, respectively, with an emission current of 8 μA. During the measurements, the base pressure of the main chamber was kept at ∼10^–10^ mbar. All the surveys and the high photon energy spectra were collected in sweep mode with a pass energy of 500 eV, while the spectra measured with Al *K*_α_ radiation were collected with a pass energy of 200 eV. For the core levels spectra, the energy step was 0.1 eV and the dwell time was fixed at 96 or 123 ms, while for the survey spectra the energy step was 0.5 eV and the dwell time was 96 ms. To isolate the contribution of the perovskite in the valence band of the powder at Al *K*_α_, we subtracted the valence bands of In foil from that of the BaZrS_3_ sample, measured with the same pass energy, dwell time and energy step size. Before subtraction, the energy calibration and intensity normalization were performed using the In 3d peaks. The peak fits were performed considering a combination of Shirley and linear background, and the peak positions and widths were found following the least-squares approximation method. The thin film S 2p_3/2_ core level peak assigned to BaZrS_3_ was measured at 161.15 eV vs Fermi. All other samples were energy calibrated by aligning the BaZrS_3_ S 2p_3/2_ core level peaks to this value, i.e., by setting the BaZrS_3_ S 2p_3/2_ peak position to 161.15 eV.

The BaZrS_3_ thin film sample was mounted in the XPS system using carbon tape, with no observed charging effects. All the spectra were collected in sweep mode and the dwell time was 96 ms. The survey was measured with 500 eV pass energy and 0.5 eV energy step size, while the core level spectra were collected with a pass energy of 300 eV and an energy step of 0.1 eV. For all the thin film measurements, Au 4f_7/2_ was employed for the energy calibration, while intensity was divided by the number of sweeps or normalized to enable a comparison with the other measurements. As calculated from the pass energy Ep, the broadening of the peaks due to the analyzer is 0.4 eV for Ep = 200 eV, 0.6 eV for Ep = 300 and 1.0 eV for Ep = 500 eV. Additionally, the resolution of the Al *K*_α_ photon source (0.25 eV) was measured to be twice as high as the resolution of Ga *K*_α_ photon source (0.5 eV).

Elemental analysis based on the XPS measurements was performed from the integrated area of the peaks normalized by the number of sweeps and by considering the cross section of the core levels with respect to the incident photon energy (more information about the cross sections of the core levels of interest at 1.487 and 9.25 keV are reported in the Supporting Information).

### Calculation Methods

For the density functional calculations, the Quantum Espresso v6.8 code^25^ was used. The calculations were performed in the full-relativistic mode. In the first round, the exchange-correlation potential was in the Perdew, Burke, and Ernzerhof (PBE) form.^26^ The band structure was calculated in the generalized gradient approximation (GGA). In the second round, the GGA exchange functional by Armiento and Kümmel (AK13)^27^ (as defined in the LibXC v5.1.6 library^28^) without the correlation part, was used instead of the PBE functional. The full-relativistic norm-conserving PBE pseudopotentials for S, Zr and Ba were generated by the code of the ONCVPSP v4.0.1 package^29^ using the input files from the SG15 database.^30^ The valence configurations defined in the pseudopotential input files were as 3s^2^3s^4^ for S, 4s^2^4p^6^5s^2^4d^2^ for Zr and 5s^2^5p^6^5d^1^6s^1^ for Ba. The plane-wave cutoff energy was set to 50 Ry. The convergence threshold for density was 1.0 × 10^–12^ Ry. The Brillouin zone was sampled by an 8 × 6 × 8 *k*-point mesh using the Monkhorst–Pack scheme.^31^ The calculations were performed for the experimental structure of BaZrS_3_^9^ without the relaxation procedure.

The size of the calculated band gap of BaZrS_3_ using the PBE functional was found to be 1.16 eV while for the AK13 functional it was 1.74 eV, the latter being in good agreement with experimental measurements.^10,13,32,33^ Furthermore, the energy positions of the shallow core levels calculated using the AK13 functional were also in better agreement with the experiments. Therefore, the results obtained with the AK13 functional are reported in our paper.

## Results and Discussion

### I Morphology and Crystal Structure

The SEM analysis performed on the powder revealed grain sizes varying from tens to hundreds of μm (see SEM image Figure S1b in the Supporting Information). The corresponding EDX revealed a global stoichiometry of: 22% Ba, 20% Zr, 50% S and 8% O, which indicates a slightly sulfur-poor composition with a minor oxygen contamination, commonly reported in thermal synthesis techniques.^13,14,17,18,34^ Nevertheless, such composition values are close to the perovskite unit formula.

**Figure 1:**
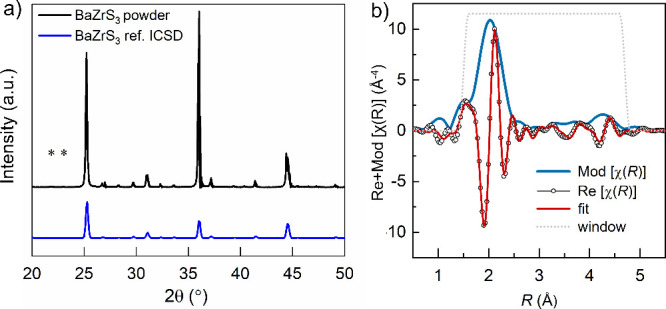
Bulk geometric structure characterization of the BaZrS_3_ powder. (a) XRD pattern of BaZrS_3_ powder compared to a reference profile of orthorhombic BaZrS_3_ (ICSD 23288); (b) Modulus of the χ(*R*) function (dark blue) representing Zr *K-*EXAFS, its real component (black open circles) and the corresponding fit (red) over the range 1.5–4.8 Å, marked by the Hanning window function (gray dotted line).

In Figure 1a, we compare the XRD profile of BaZrS_3_ powder to the reported reference BaZrS_3_ (ICSD 23288). The figure shows a clear matching of the prominent Bragg peak positions, namely 2θ = 25.2°, 31.0°, 36.0° and 44.4°, proving the successful formation of the crystalline BaZrS_3_. These reflections can be indexed to (121), (220), (040) and (042) planes, respectively, characteristic of *Pnma* symmetry observed for the BaZrS_3_ powder. We notice minor additional peaks “*” (2θ = 21.9° and 23.0°) which can most likely be assigned to Ba–S–O compounds, and not to Ruddleson-Popper phases. Considering the substoichiometry of S in BaZrS_3_ powder and the correspondence between the XRD BaZrS_3_ reference profile and the data, it can be deduced that these contributions are impurities related to undesired surface oxidation, rather than originating from e.g., BaZrS_3_–BaZrO_3_ alloys. Since the space group *Pnma* is orthorhombic with the lattice constants *a* ≠ *b* ≠ *c*, one needs to apply Bragg’s law to multiple reflections from the XRD profile to independently estimate the values of *a*, *b* and *c*. Using the three major reflections, (121), (220) and (040), we arrive at the lattice constants: *a* = 7.053 Å, *b* = 9.973 Å, *c* = 7.074 Å, which are well in agreement with the values reported in the literature (*a* = 7.0599(8) Å, *b* = 9.9813(17) Å, *c* = 7.0251(12) Å).^9,35^ Further employing the Debye–Scherrer’s law to the three Bragg reflections, we find an average crystallite size of ∼43 nm. A similar analysis of the BaZrS_3_ thin films yields unit cell dimensions of *a* = 7.051 Å, *b* = 9.957 Å, *c* = 7.048 Å, rather similar to the unit cell dimensions of the powder sample, but a smaller average crystallite size of ∼22 nm, as already reported.^13^ Further detailed characterization of the polycrystalline thin film can be found in previous reports.^10,18^ The powder and thin films of BaZrS_3_, although grown using very different synthesis strategies, achieve the same orthorhombic structure with rather comparable unit cell dimensions, suggesting the orthorhombic symmetry to be energetically favored over other higher symmetry perovskite structures. The duration of the thermal process, i.e., 1 min for the thin films, and several weeks for the powder, respectively, does not seem to have a major impact on the lattice dimensions. It mainly affects the grain growth, forming significantly larger grains for powder (>10 μm as qualitatively shown by the SEM image in Figure S1b in the Supporting Information) compared to thin films (∼100 nm), as evidenced by electron microscopy analyses.^13^

Table 1 compiles the morphological and geometric structure information for the powder in terms of crystallite sizes, unit cell dimensions, Zr–S interatomic distances and Zr–S disorder (σ^2^), and compares it to the reported parameters for thin film.^21^ From Zr *K*-edge XAS measurements it is possible to extract the modulus of χ(*R*), which illustrates the average geometric distribution of the near neighboring atoms around Zr atoms in real space (Figure 1b, blue). For the powder, our first shell EXAFS data analysis yields average Zr–S bond distances ∼2.54 Å, similar to Zr–S bond distances (2.53 Å) calculated from the reference XRD data.^9^ Higher shell EXAFS data analysis reveals two sets of Zr–Ba interatomic distances ranging from 4.08 to 4.39 Å, and Zr–Zr interatomic distances of 4.84 Å (Table S1 in the Supporting Information). The extracted pseudo-Debye–Waller factor (σ^2^), accounting for the Zr–S bond variance, amounts to ∼0.006 Å^2^. Such a low σ^2^ value is suggestive of the high crystallinity of the BaZrS_3_ powder. A lower variance of the Zr–S bond distance is estimated for the powder as compared to the film. Overall, we find the parameters in Table 1 to be rather comparable, except for the higher crystallinity of the powder, as evidenced by the bigger crystallite size and lower σ^2^ for the Zr–S bond for the powder as compared to the film. Nonetheless, this comparison confirms the good quality of the thin film. The average crystallite size for powder is still small, indicative of the rather high activation energy barrier for BaZrS_3_ formation and growth of crystallites, i.e., single domains. A longer thermal process facilitates several such domains to join along the crystallite boundaries and form large polycrystalline grains instead of forming larger crystallites. In contrast, rapid annealing in the films limits this process to a much smaller grain size.

**Table 1:** Comparison of Bulk Long- (XRD) and Short-Range (EXAFS) Geometric Structural Parameters between the BaZrS_3_ Powder and Thin Films in Terms of: Average Crystallite Sizes, Lattice Parameters, Zr–S Bond Lengths and Zr–S Bond Disorder^a^

	XRD		EXAFS	
	crystallite size (nm)	lattice parameters (Å)	Zr–S distance (Å)	Zr–S disorder σ^2^ (Å^2^)
powder	43 ± 6	*a* = 7.053 ± 0.009	2.539 ± 0.004	0.0059 ± 5 × 10^–4^
		*b* = 9.973 ± 0.027	2.532 ± 0.007*	
		*c* = 7.0735 ± 0.029		
thin film	22 ± 1	*a* = 7.051 ± 0.019	2.546 ± 0.006	0.0066 ± 3.1 × 10^–4^
		*b* = 9.957 ± 0.055		
		*c* = 7.048 ± 0.058		

a Parameters marked with * are estimated from BaZrS_3_ reference (ICSD 23288)^9^ from Figure 1a.

### II Electronic Structure

#### Core Level Photoelectron Spectroscopy

**Figure 2:**
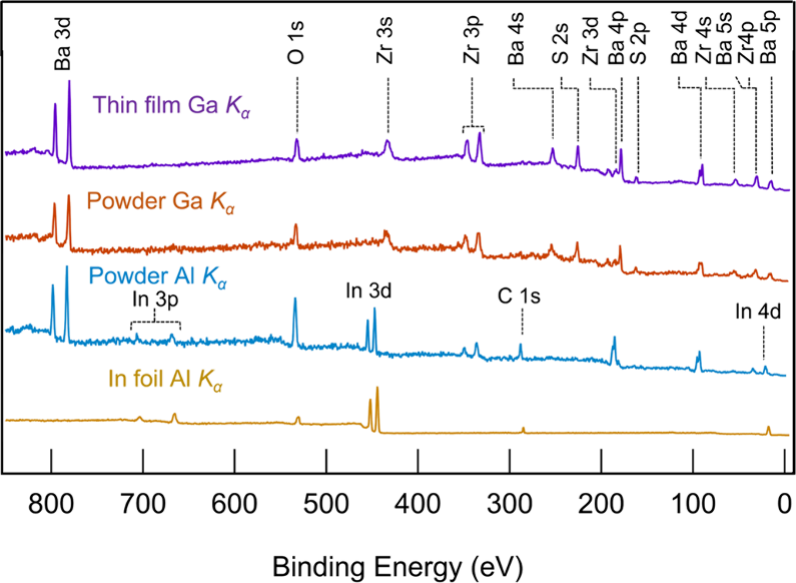
Photoelectron spectroscopy survey scans of the BaZrS_3_ powder (blue, orange) and thin film (purple) measured with Al *K*_α_ and Ga *K*_α_, showing different core level peaks indexed. For comparison, the survey scan of Indium foil measured with Al *K*_α_ is reported (yellow).

Figure 2 shows survey scans measured for the bare In foil, BaZrS_3_ powder on In foil and BaZrS_3_ thin film, collected using the different photon energy sources. All three surveys on BaZrS_3_ show core level peaks from Ba, Zr, S, O and C, while the low photon energy survey spectrum of BaZrS_3_ powder additionally contains the peaks of In. The substrate signals do not interfere with the main core levels of interest from the BaZrS_3_ sample (Ba 3d, Zr 3p, Z 3d and S 2p), which remain distinctively visible. Overlap between signals originating from the BaZrS_3_ sample and In foil occurs for O 1s, C 1s and the valence band spectrum. Consequently, the BaZrS_3_ valence band spectrum of the low photon energy measurement of the powder was obtained by subtracting the valence band spectrum of the bare In foil. The survey scans for both samples, measured with Ga *K*_α_, exhibit signatures of all relevant core levels of BaZrS_3_ with a clear O 1s signal, but no clear C 1s signal. The probing depth with Ga *K*_α_ is therefore high enough for both powder and thin film samples to suppress the signal coming from adventitious C at the sample surface, while still detecting the presence of O deeper into the material.

**Figure 3:**
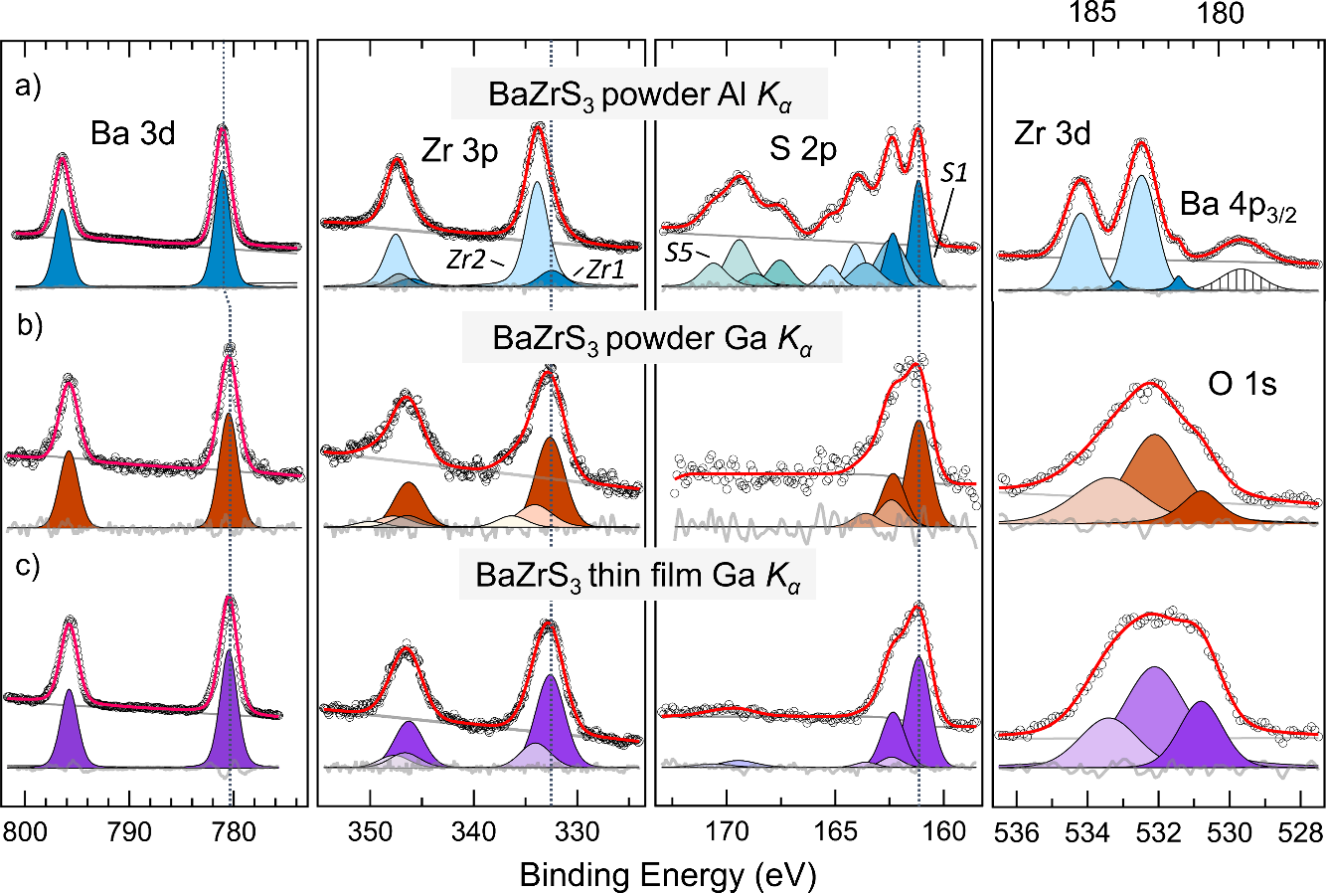
(a) Core level XPS spectra of Ba 3d, Zr 3p, S 2p and Zr 3d measured at low photon energy (Al *K*_α_: 1.487 keV) for BaZrS_3_ powder, (b) Core level XPS spectra of Ba 3d, Zr 3p, S 2p and O 1s measured at high photon energy (Ga *K*_α_: 9.25 keV) BaZrS_3_ powder, and (c) BaZrS_3_ thin film. The spectra are energy calibrated vs Fermi of the thin film, calculating the binding energy of S1 S 2p_3/2_ at 161.15 eV.

Figure 3 shows the core levels of Ba 3d, Zr 3p, S 2p for the powder sample at low – Al *K*_α_ (Figure 3a) - and high – Ga *K*_α_ (Figure 3b) – photon energies, and for the thin film sample at Ga *K*_α_ (Figure 3c). In addition, the corresponding Zr 3d core level is shown for the powder at low photon energy (Figure 3a, top right panel), while the O 1s core level is shown for the high photon energy measurements of both systems (Figure 3b, middle right and Figure 3c, bottom right panels). The Ba 3d peaks can be fitted with one spin–orbit split state with a fixed intensity ratio of 3:2 for all measurements and with the spin-orbit splitting energy of about 15.3 eV, consistent with the value of 15.33 eV reported in the literature.^36,37^ The binding energy of Ba 3d_5/2_ amounts to 780.50 eV for both the powder and thin film samples when measured with Ga *K*_α_, while a value of 781.10 eV is obtained for the powder when measured using Al *K*_α_, as marked by a vertical dotted line in Figure 3. The peak widths for the spin–orbit split Ba 3d states are 2.13 and 1.96 eV for the powder and the thin film sample, respectively, as measured by Ga *K*_α_, while it is slightly lower for the powder when measured by Al *K*_α_ (1.80 eV). This difference is in large part accounted for by the contribution to the resolution expected from the different pass energies used for these measurements (see the Methods/Experimental Section).

Two distinct contributions to the Zr 3d peaks are discernible with the Zr 3d_5/2_ components occurring at 181.60 and 183.08 eV when measuring the powder using Al *K*_α_ (Figure 3a, right panel). For Ga *K*_α_ (Figure 3b,c), the peaks of Zr 3d (185–180 eV) suffer from a higher noise level in the signal resulting from the lower cross section of Zr 3d compared to Ba 4p_3/2_, and the complex structure of satellite peaks instead of the sharp Ba 4p_1/2_ peak.^38^ For this reason, the chemical environment of zirconium was mainly investigated through the core level Zr 3p at high photon energy. For the three measurements of Zr 3p, the fits were executed by constraining the binding energy separation value between the chemical species to be the same for all the spectra and aligning the peak positions at the same binding energy. Additionally, the Zr 3p_1/2_ and Zr 3p_3/2_ peaks were deconvoluted by keeping the energy difference to 13.66 eV and fixing the intensity ratio as 2:1, defined by the spin–orbit splitting. A background peak (Figure 3, gray) caused by a shakeup transition was also considered for determining the correct intensity ratio.^39^

For the measurements at Al *K*_α_, the positions of the Zr 3p_3/2_ peaks are obtained at 332.44 eV and 333.86 eV (labeled as Zr1 and Zr2 respectively in Figure 3), with an energy difference (Zr 3d_5/2_ - Zr 3p_3/2_) of 150.8 eV for both species. For this photon energy, the FWHM is 2.9 eV for Zr1 and 2.6 eV for Zr2. The corresponding Ga *K*_α_ spectra show Zr 3p_3/2_ peaks at 332.64 eV and 334.11 eV for the powder, and at 332.62 eV (Zr1) and 334.05 eV (Zr2) for the thin film, respectively. In addition, the powder contains a third peak of Zr 3p_3/2_ at 336.33 eV. The widths for the Zr 3p_3/2_ peaks measured by Ga *K*_α_ are 3.3 eV for Zr1 and 4.0 eV for Zr2 for the powder; corresponding peak widths for the thin film are 3.3 eV (Zr1) and 3.4 eV (Zr2), respectively. Focusing on the two common zirconium species Zr1 and Zr2, we notice that their relative intensities change on probing from low to high photon energies: with Al *K*_α_, the predominant species is Zr2, while with Ga *K*_α_ Zr1 becomes more intense. Considering that the probing depth of the XPS increases from a few nm with Al *K*_α_ to 10–15 nm with Ga *K*_α_, we can conclude that Zr2 corresponds to the oxidized zirconium present in the surface region (most probably ZrO_2_ or BaZrO_3_), whereas Zr1 can likely be assigned to the perovskite structure BaZrS_3_ in the bulk.

Our microscopy studies^13^ support the presence of Zr-oxide species in the thin film, even when measured with Ga *K*_α_. From the STEM analysis of the thin film combined with the elemental map distribution, we notice that the O-rich phases are linked to the Zr-rich phases, indicating the dispersion of ZrO_*x*_ species throughout the bulk of the sample. Such secondary phases could form during the nucleation of the BaZrS_3_ grains, which leads to the aggregation of O around Zr, a process also likely to be aided by the presence of pinholes and cracks in the thin film. In contrast, the powder is fabricated in a sealed ampule, and it is less likely that oxygen atoms are absorbed from the atmosphere during the annealing process. Rather, a more plausible scenario is that some oxygen is already trapped in the sealed ampule which reacts during annealing, or the oxidation process occurs after exposure to ambient conditions. However, considering the very different synthesis process for the two samples which reveals very similar XPS spectra, we believe that the oxygen contamination probed at the surface occurs upon exposure to air.

As clearly observed in Figure 3, the S 2p spectrum exhibits a multicomponent structure, ranging from two to five distinguishable components (labeled as S1 with the lowest binding energy peak, marked by dotted vertical line in Figure 3, to S5 with the highest binding energy peak). The most complex and resolved S 2p spectrum of the Al *K*_α_ was deconvoluted, and then the results were subsequently employed to fit the Ga *K*_α_ S 2p spectra. During the fitting procedure, the intensity ratio of the S 2p_3/2_ and S 2p_1/2_ was set to 2:1, and the energy splitting between the two S 2p peaks was set to 1.18 eV. The high complexity of the S 2p peaks measured at low photon energy can be associated with a varied chemical environment of the sulfur within the film surface region. From the fit, the component S 2p_3/2_ corresponding to S1 is observed at 161.15 eV, while components S2 and S3 appear at 162.41 and 164.06 eV, respectively. Two more distinct states S4 and S5 can be observed at 167.55 and 169.41 eV, which can be attributed to the oxidized sulfur compounds of BaSO_3_ and BaSO_4_.^40^ The results from the high photon energy, which probes deeper into the sample, can also be fit with S1 and S2 having the same energy positions for both the powder and the thin film samples, i.e., S 2p_3/2_ of S1 at 161.15 eV and S2 at 162.41 eV. In the case of the thin film (Figure 3c), an adventitious sulfur component linked to sulfate is found at 169.46 eV. As for the other core levels, the S 2p peaks measured using the Al *K*_α_ source show narrower features than the ones measured by the Ga *K*_α_ radiation. This effect stems from the experimental requirement of a higher pass energy (and thus higher transmission) when using the Ga *K*_α_ source to compensate for the lower core level photoemission intensity. This explains why for Al *K*_α_, the lowest binding energy peak shows an FHWM of 0.84 eV, while for Ga *K*_α_ the FWHM is ∼1.2 eV for both the measurements of the powder and the thin film. Unlike the zirconium features, the sulfur peaks of the powder show stronger dynamicity when the probing depth increases with the hard X-rays in XPS. In fact, from a five-species manifold of sulfur at Al *K*_α_, only two signatures could be detected with the Ga *K*_α_ source. As indicated previously, S 2p peaks can be classified into two subgroups: the higher binding energy peaks related to the sulfur oxides (172–166 eV), and the lower binding energy peaks (165–161 eV). Validation of the oxidized S species is given by the binding energy difference Ba 3d_5/2_ – S5 S 2p_3/2_ of 611.69 eV, comparable to the literature value of 611.5 eV for the barium sulfate BaSO_4_.^40^ The lower energy sulfur peaks S4 are assigned to the barium sulfite BaSO_3_, where Ba 3d_5/2_ – S4 S 2p_3/2_ is 613.55 eV. The binding energy difference between the S5 and S4 S 2p_3/2_ is ∼1.9 eV, consistent with the energy difference expected for sulfate–sulfite species.^41^ One can clearly observe that the S3 and S2 signals decrease significantly in intensity relative to S1 in hard X-ray measurements as compared to soft X-rays. This observation hints at both S2 and S3 features being related to surface terminated sulfurs, with S3 from the bonds S–C or S–H and S2 from the Ba–Zr–S-C.^42^ Finally, the S1 peak corresponding to the lowest binding energy peak in the S 2p spectra appears for all three measurements, increasing in intensity with higher probing energy, suggesting its close relation to the sulfur peak of the perovskite structure BaZrS_3_.

O 1s was measured for both BaZrS_3_ powder and the thin film at high photon energy. In both cases, the data was fitted with three peaks at 530.80 eV, 532.11 and 533.41 eV, assigned to Zr bound to O, BaSO_4_ and chemisorbed O, respectively, with the proportion of the different O species differing between the powder and the thin film. Similar results were obtained in the paper by Mukherjee et al.,^21^ where varied surface oxidation effects induced at different annealing temperatures led to varying ratios of the component chemical species.

A range of O products is observed in the material up to 15 nm, as confirmed by the presence of sulfur oxides and a Zr–O-rich phase (shown in Figure 3), consistent with our previous observations.^13^ For both samples, we notice that the distribution of the oxide species varies along the sample depth: the sulfur oxides BaSO_3_ and BaSO_4_ are limited to the first 1–2 nm of the surface, while the ZrO_*x*_ species extend deeper than 10 nm from the sample-air interface. The oxidation variability within the first 10 nm of the perovskite surface might be detrimental to device integration due to the typically high resistance of the oxide compounds.

In contrast to the sulfur and the zirconium spectra, the Ba 3d spectra do not reveal any specific changes in the bonding environment, assuming a predominant oxidation state +2, which includes contributions from chemical species besides the perovskite. However, considering S1 as the perovskite peak at the same binding energy for all three sets of measurements, the position of Ba 3d_5/2_ varies slightly with the photon energy: 781.10 eV for Al *K*_α_, and at 780.50 for Ga *K*_α_. At high photon energy, the XPS measurements reveal a diminished presence of mixed compound species (e.g., BaSO_4_) as compared to the Al *K*_α_, which may contribute to the shift of the Ba 3d_5/2_ peak toward lower binding energy values. Other oxidation compounds involving barium, such as BaO_2_, cannot be ruled out. A diagram of the electronic structures compared for the two photon energies are reported at the end of the paper.

As opposed to the powder sample, where energy calibration was performed internally and only relative energy differences could be considered, all the peaks for the thin film were energy-calibrated according to the Au 4f_7/2_ peak position at 84.0 eV. Table 2 reports the peak positions of selected core levels representative of all the elements present in BaZrS_3_, where the lowest binding energy peaks of Zr1 Zr 3p_3/2_ and S1 S 2p_3/2_ are listed.

**Table 2:** Core Level Binding Energy Values of Different Constituent Elements for the BaZrS_3_ Thin Film Measured at Ga *K*_α_, Calibrated against the Fermi Level

	Ba 3d_5/2_ (eV)	Zr1 Zr 3p_3/2_ (eV)	S1 S 2p_3/2_ (eV)
binding energy vs Fermi	780.50	332.62	161.15

Table 3 reports the binding energy difference between the peaks of Ba 3d_5/2_, Zr1 Zr 3p_3/2_ and S1 S 2p_3/2_, based on the peak positions obtained from the fits to the core levels measured with Al *K*_α_ and Ga *K*_α_, and compares the values for both powder and thin film. We notice that the energy differences between the peaks collected at Ga *K*_α_ are rather similar and within the experimental uncertainty limits. This observation can be extended to the case of Al *K*_α_ for the binding energy offset between Zr and S, with the exception of the values accounting for the Ba 3d_5/2_ which resides at higher binding energy values when measured with Al *K*_α_ (see discussion above). These findings are in agreement with our previous photoemission report on surfaces of BaZrS_3_ thin films.^21^

**Table 3:** Binding Energy Offsets between the Barium, Zirconium and Sulfur Peaks for Our Model Systems of BaZrS_3_^a^

	Ba 3d_5/2_ – S1 S 2p_3/2_ (eV)	Zr1 Zr 3p_3/2_ – S1 S 2p_3/2_ (eV)	Ba 3d_5/2_ – Zr1 Zr 3p_3/2_ (eV)
Al *K*_α_ XPS – powder	619.95	171.29	448.66
Ga *K*_α_ XPS - powder	619.35	171.49	447.86
Ga *K*_α_ XPS - thin film	619.35	171.49	447.88

a Calculated from the fitted peak positions of Ba 3d_5/2_, and the lowest binding energy peaks position of Zr1 Zr 3p_3/2_ and S1 S 2p_3/2_ for the three cases investigated through XPS.

Averaging the offset values Zr1 - S1 for all the measurements reported in Table 3, the mean binding energy difference (Zr1 3p_3/2_ – S1 2p_3/2_) for the BaZrS_3_ perovskite amounts to 171.42 eV.

Table 4 provides elemental composition analyses obtained from our photoemission measurements performed for the BaZrS_3_ powder and thin films, in comparison to the atomic percentages given by EDX data. The elemental composition from the XPS spectra was obtained through the sum of the areas of all the fitted peaks of Ba 3d_5/2_, Zr 3p_3/2_, S 2p_3/2_ and O 1s weighted by their cross section at 9.25 keV (the calculation of the cross sections at 9.25 keV is reported in the Supporting Information), and taking into account the number of sweeps and the acquisition time. It is interesting to note that the EDX compositional analyses on the thin film and the powder match, although the synthesis processes are completely different. The presence of O in the bulk of the thin film can be explained by the ToF-ERDA measurements by Comparotto et al.,^13^ which revealed significant surface oxidation in the first few nanometers of the thin film, and the bulk containing clusters of amorphous Zr–O which were not captured by XRD. In the powder, the presence of atomic O is not clearly unraveled, whether it is associated with only surface or also has contributions from the bulk. The dissimilarity in the values reported by EDX and the XPS analysis for both samples is significant. This is not surprising, considering the appreciably higher bulk sensitivity of EDX, probing up to several micrometers into the material, while XPS probes only the first few nm from the material surface. When the composition is evaluated by XPS (Table 4), the atomic percentage reveals the partial substitution of sulfur by oxygen. For the powder sample the ratio of O/S is 1.45, while for the thin film it is 0.94. This is in sharp contrast to the much lower O/S ratio (around 0.16) for both powder and films obtained from the EDX data, demonstrating that the oxygen is mainly present in the surface for at least 10 nm, and the true perovskite without impurities resides only deeper in the sample. The elemental estimation from spectroscopy displays a lower concentration of S ([S]/([Ba] + [Zr] + [S) < 0.6) compared to the EDX analyses. This is primarily related to S being substituted by O (i.e., [S]/[S] + [O] < 1), an effect that is dominant in the first ten nanometers of the surface. We note here that the replacement of S by O simply results from undesired surface reactions, and not necessarily random anionic site substitution of S by O in the BaZrS_3_ lattice, as already negated by our previously published structural data.^21^ The O substitution at S sites in BaZrS_3_ has been suggested to modify material properties including potentially yielding ferroelectric behavior.^43^ In our case, it appears that O preferentially existed in ZrO_*x*_ clusters. The influence of these in material properties is a question for further research. Probing from low to high photon energy for the powder sample by XPS, we find that the ratio ([Ba]/(Ba] + [Zr]) increases and gets closer to the stoichiometric value, and [S]/([Ba] + [Zr] + [S]) ratio approaches the value obtained from EDX analyses. Irrespective of the relative amounts of the atomic species, at high photon energy the spectra of Ba 3d, Zr 3p and S 2p overlap completely (Figure 3). Our observations indicate that oxidation is difficult to avoid during the preparation of our BaZrS_3_ samples. We note that the synthesis methods used in this study have been widely used to prepare oxygen-free chalcogenides of other types, and yet for this material, both synthesis procedures resulted in the same superficial oxygen-containing species. It is still not clear whether these arose due to a particular sensitivity of the material to trace oxygen during high temperature processing, or due to spontaneous oxidation of BaZrS_3_ surfaces upon air exposure, or due to a combination of both. Because of the resulting oxidation species on the surface, it is necessary to clean at least the top 10 nm of BaZrS_3_ samples before proceeding to the device integration. This may be done with chemical or physical methods that remove the adventitious species, or by improving the control of the surface reactivity during synthesis.

**Table 4:** Composition of the Powder and the Thin Film Sample, Expressed as Ratios of Elemental Concentrations Estimated from EDX and XPS Techniques^a^

	[Ba]/([Ba] + [Zr])	[S]/([Ba] + [Zr] + [S])	[O]/([Ba] + [Zr] + [S] + [O])	[S]/([S] + [O])
EDX - powder	0.52	0.55	0.08	0.86
Al *K*_α_ XPS – powder	0.29	0.23	*	*
Ga *K*_α_ XPS - powder	0.58	0.49	0.42	0.41
EDX - thin film	0.50	0.54	0.08	0.86
Ga *K*_α_ XPS - thin film	0.57	0.48	0.31	0.52
stoichiometric	0.50	0.60	0.00	1.00

a The symbol * denotes cases where the compositional ratio is not applicable.

#### Valence Band Photoemission and DFT Calculations

**Figure 4:**
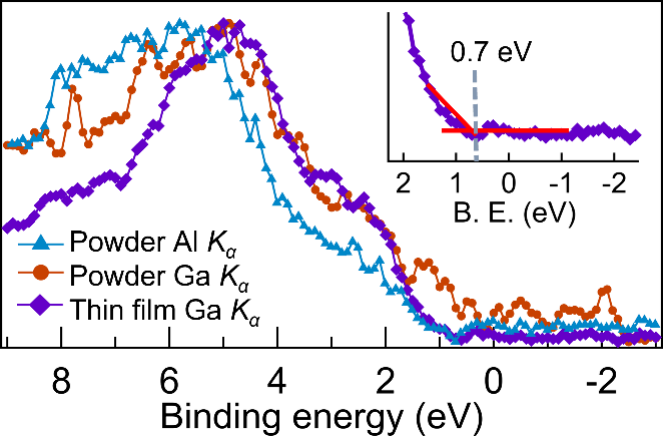
Valence band spectra acquired for BaZrS_3_ powder obtained with Al *K*_α_ photon energy (blue triangles), Ga *K*_α_ photon energy (orange circles), and for the thin film with Ga *K*_α_ photon energy (purple diamonds). All the spectra are calibrated through the S 2p_3/2_ at 161.15 eV. Intensities are normalized for comparison. Inset: enlarged view of the valence band edge of BaZrS_3_ thin film measured with Ga *K*_α_, showing the position of the VBM for the BaZrS_3_ thin film determined from the intersection of red lines (energy position marked by vertical dashed gray line).

Another crucial feature of the electronic structure that can be studied and compared among the samples is the valence band, which contains information about the density of states of the hybridized orbitals for a given system, consequently defining the samples’ properties. The valence band spectra for the two systems measured with soft (Al *K*_α_) and hard X-rays (Ga *K*_α_) are shown in Figure 4. In the figure, all three valence bands were normalized to the main feature at around 5 eV, with energies calibrated against the Fermi level of the thin film, as described in the Methods/Experimental Section. Qualitatively, the measurements acquired at high photon energy exhibit the same shoulder between 0 and 4 eV, and they have a narrow feature around 5 eV. On the other hand, the valence band spectrum measured with lower photon energy shows similar, albeit broader features, where the low binding energy shoulder has smaller intensity and the feature at 5 eV prevails in the valence band spectrum.

**Figure 5:**
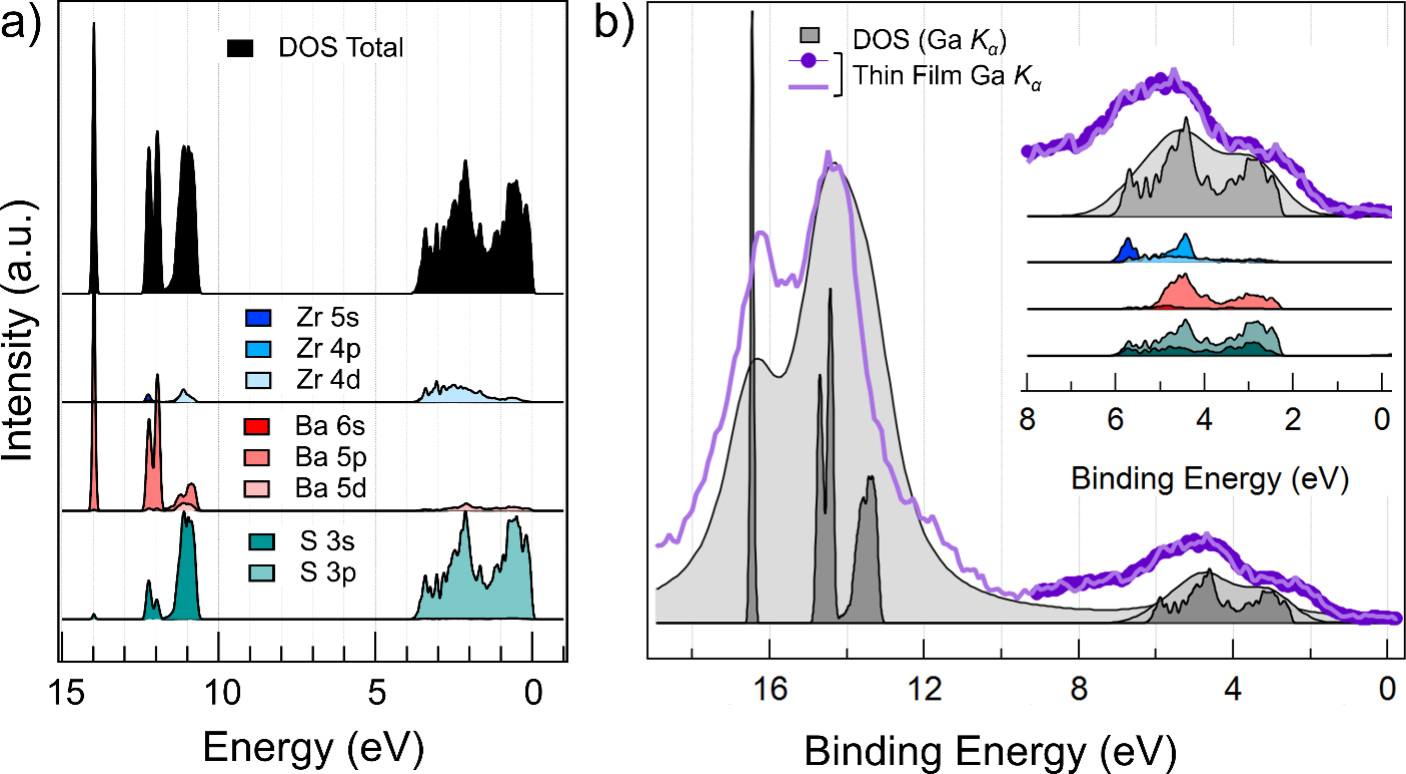
(a) Orbital-resolved projected density of states for orthorhombic BaZrS_3_ (*Pnma* symmetry). (b) Comparison of the experimental valence bands of thin film sample (photon energy: 9.25 keV) with the theoretical contributions of single atoms, weighted by the cross sections at 9.25 keV. The density of states in panel (b) are energy calibrated by Au 4f_7/2_ at 84.0 eV.

Figure 5a shows the orbital-resolved projected density of states of the BaZrS_3_ matrix obtained from DFT calculations, displayed to the valence band maximum at energy 0 eV, which allows us to identify the orbital contributions of the different constituent elements of BaZrS_3_ in the valence band. It can be clearly seen that the theoretical DOS in the range 0–5 eV results predominantly from the 3p states of the S atoms and 4d states of the Zr atoms, in accordance with previous reports.^10,12,44^ Moving to the energy range 10–15 eV, we find that the total DOS results from the hybridization of the p-states of barium with the s-states of sulfur, explaining the difficulty of fitting the peaks with the 2:1 ratio for the intensity of Ba 5p in the experimental spectra. It is clear from the DFT calculations that the band edges are populated primarily by contributions from the orbitals of Zr and S, facilitating the optical transition between the S 3p states in the valence band to the transition metal d states in the conduction band, and consequently, leading to a strong band edge light absorption. The orbitals of Ba, on the other hand, are more deep-seated in energies. This follows from the perovskite structure, where Ba resides at the A-site assuming a larger cuboctahedral volume (Ba–S_12_, i.e., with 12 sulfur atoms) and consequently showing weaker hybridization effects compared to a much smaller Zr–S_6_ octahedral volume. Some minor mixing of Ba states is promoted by a large spatial distribution of Ba atoms in orthorhombic symmetry, with Zr–Ba interatomic distances spanning from 4.08 to 4.39 Å as extracted from EXAFS analysis, which has an insignificant effect on light absorption. The population of Zr d orbitals in the conduction band edge and chalcogen p orbitals in the valence band edge, would both be sensitive to the degree of cooperative tilting of adjacent Zr–S_6_ octahedral units along the three crystallographic axes. Our EXAFS analysis indeed reveals distorted Zr–S–Zr bond angles of 144–147° in the case of both powder and thin film, slightly lower than angles reported from XRD (159.477 ± 0.27°).^9^ The commonly observed difference between the two structural probes^45,46^ stems from inaccuracies in accounting for multiple scattering contributions on scattering amplitude and phase functions.^47^ Nonetheless, it shows that these octahedral rotations bring adjacent Zr sites closer, consequently lowering the orbital overlap and forming narrower bands. Notably, the conduction band also includes some contributions from S 3p states, expected from Zr–S bonds having some covalent character. The interplay between Zr–S covalency and Zr–S–Zr angles are crucial factors that determine the band gap of BaZrS_3_ to be ∼1.7–1.8 eV, making it a semiconductor, unlike the chalcogenide analogue BaZrO_3_, where higher ionicity of Zr–O bonds leads to less hybridization and widening the gap to 3.9 eV.^44^

For a proper comparison of the calculated DOS with the experimental XPS spectra, the calculated DOS needs to be weighted by the respective orbital cross sections for the probing XPS energy. We find that the valence band of BaZrS_3_ measured at Al *K*_α_ (Figure S2 in the Supporting Information) reproduces the theoretical DOS quite well. In the present study, we focus on the valence band spectrum recorded with Ga *K*_α_ due to the suppressed surface contributions, rather than the Al *K*_α_ measurements. Therefore, Figure 5b reports the theoretical DOS weighted by the XPS cross section of the Ga *K*_α_ energy, as compared to the experimental spectra. From Figure 5b we notice that the valence band features acquired at Ga *K*_α_ contain the contributions from all the perovskite atoms. In particular, the Ba 5p states are enhanced compared to the S 3p and Zr 4d, making the low binding energy shoulder (at around 2 eV) attain comparable intensity as the one at 5 eV. Applying Lorentzian and Gaussian broadening to the theoretical results, the two features evolve into the displayed gray area, attaining a similar energy profile to our experimentally obtained valence band spectrum at Ga *K*_α_, both for the thin film and the powder.

With respect to the theoretical DOS weighted to the cross sections, the valence band spectra generally show broader features and a higher background, which can be explained by scattering effects in the photoemission process, as well as the presence of mixed compounds and impurity atoms. As seen in the theoretical results, the states of S and Zr contribute to the valence band shape, as displayed when BaZrS_3_ samples are measured with soft and hard X-rays. From our core level measurements of S 2p and Zr 3p shown in Figure 3, we noticed the presence of mixed compounds attributed to Zr–O and Ba–S–O residing in the first 1–2 nm of the surface, and hence evidenced especially when measured with Al *K*_α_ radiation. Their presence is most probably reflected in the broadening of the valence band spectrum up to 8 eV, with the addition of the oxygen feature noticeable by the peak at around 7 eV, corresponding to the O 2p (Figure 5 and Figure S2 in the Supporting Information).^48,49^ On the other hand, the valence band spectra measured at Ga *K*_α_ (Figure 5b) are narrower, in particular for the thin film, confirming the low contribution of oxidized species to these spectra.

Determining the valence band maximum relative to the Fermi level holds significance in understanding interfacial energetics and in the construction of the energy diagram of materials when put in contact. In our XPS measurements, energy calibration against Fermi was feasible only for the thin film probed by Ga *K*_α_ radiation. Using a linear intersection between the valence band edge feature and the baseline, we could determine the VBM to be at 0.7 eV vs Fermi level (see Figure 4, inset). The measurements on the polycrystalline powder were aided by the neutralizer, making it difficult to calibrate the spectra in an appropriate way. Therefore, the measurements for powder were energy calibrated by assigning the S 2p (S1) binding energy position at 161.15 eV at all probing energies. Neglecting the valence band of the powder at Ga *K*_α_ because of the low signal-to-noise ratio, we notice that the VBM for the powder at Al *K*_α_ appears at the same binding energy position as for the Ga *K*_α_ spectrum for the thin film (see Figure 5). From this, we can suggest that the secondary oxide phases do not interfere with the frontier valence band region (from 0 to 4 eV) and specifically not with the valence band maximum. These typically large-gap oxides, therefore, seem to coexist with BaZrS_3_ and remain mostly confined to the surface, as opposed to forming alloys and increasing the overall band gap.

The position of the VBM relative to the Fermi level determines the type of a given semiconductor. Considering the reported band gap of 1.84 eV as obtained from photoluminescence measurements for this specific thin film,^13^ the valence band measurements indicate that the Fermi level is closer to the VBM, rather than to the conduction band minimum, therefore suggesting weak p-type doping. This result is in agreement with Han et al.,^35^ even though the conductivity type of BaZrS_3_ is still a debated topic. For example, Wei et al.^6^ found that BaZrS_3_ thin films are of n-type, likely due to the sulfur vacancies. Clearly, the nature of doping is strongly influenced by synthesis strategies and further research is necessary in this regard.

**Figure 6:**
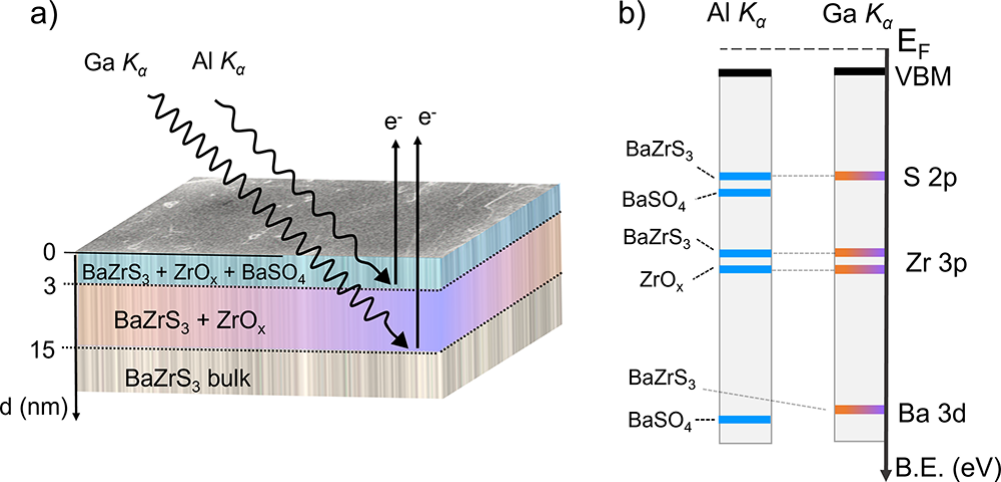
Schematic representation of (a) phase distributions, (b) core level positions and valence band maxima for BaZrS_3_ samples measured at Al *K*_α_ and Ga *K*_α_ radiations. The solid lines represent the core level binding energy positions (blue for Al *K*_α_ and orange-to-purple for Ga *K*_α_) in an increasing binding energy scale with 0 at the Fermi level (*E*_F_).

Figure 6 schematically summarizes the phase distributions along the BaZrS_3_ surface (Figure 6a) and the results of the BaZrS_3_ electronic structure (Figure 6b), as investigated in this paper. In the figure, the core levels are represented by solid lines in an increasing binding energy scale with 0 at the Fermi level (*E*_F_). As discussed earlier, the S and Zr peaks at Al *K*_α_ and Ga *K*_α_ are at the same binding energy position, with the exception of Ba 3d. Differently from the bulk studies, our approach highlights the importance of proper identification of all core level spectra and the valence band to arrive at a comprehensive understanding of the electronic structure of BaZrS_3_, and determine the type of semiconductor achieved through different synthesis strategies.

## Conclusions

Our study concerns the investigation of chalcogenide perovskites BaZrS_3_ samples in the form of powder and thin film with regard to their electronic, chemical and geometrical properties. After ensuring good material quality of both systems in terms of bulk geometric structure and stoichiometry, we focused on the study of the surface composition using photoelectron spectroscopy at two photon energies Al *K*_α_ and Ga *K*_α_, validating the material chemistry at different probing depths. While the more surface sensitive Al *K*_α_ on the powder shows stronger signals from oxidation species, the more bulk sensitive Ga *K*_α_ measurements on the two samples reveal a significant resemblance in the chemical environments, related to the BaZrS_3_ perovskite. For all the XPS measurements, only one main peak of Ba 3d could be resolved, obscuring the detailed chemistry of the barium atom. Conversely, the analysis of the zirconium and sulfur spectra reveals that the oxides related to zirconium extend more than 10 nm toward the bulk of the samples, while oxysulfide compounds are confined only to the first 1–2 nm from the sample surface. Except for the persistent surface oxidation effects, the BaZrS_3_ spectra measured with Ga *K*_α_ source on both sample types were cleaner and allowed us to identify the peaks of the BaZrS_3_ matrix. The spectra of valence bands measured matched well with the theoretical DOS, substantiating how bonding characteristics are correlated to the orthorhombic structure maintained through the angular −Zr–S-Zr– inorganic network. At high photon energy, we find the valence band spectra to be contributed strongly by the orbitals of all atoms S, Zr, and Ba, enhanced by the higher cross section from the photoelectric process. Finally, our analysis of the valence band edge indicates that the presence of the oxides does not contribute to the position of the valence band maximum with respect to the Fermi level, thereby elucidating the doping character of the BaZrS_3_ semiconductor. The above observations allow us to achieve a comprehensive insight into the electronic landscape of BaZrS_3_, and such a strategy could be extended to other members of the chalcogenide perovskite group in general to harness their full potential as photovoltaic materials.

## Supporting Information Available

Details about: 1. EXAFS parameters, 2. Powder, 3. Cross sections at 9.25 keV, 4. DOS and experimental valence band at Al *K*_α_. Table S1: Parameters extracted from higher NN fits to Zr *K*-XAS data for the BaZrS_3_ powder and thin film. Tables S2 and S3: Cross section values for the selected core levels and valence band states calculated at 9.25 keV. Figure S1: SEM images of the BaZrS_3_ powder. Figure S2: Agreement between density of states weighted on the cross section and experimental valence band at Al *K*_α_. (PDF)

## Abbreviations

XPS: X-ray photoelectron spectroscopy
HAXPES: hard X-ray photoelectron spectroscopy
XAS: X-ray absorption spectroscopy
EXAFS: extended X-ray absorption fine structure
XRD: X-ray diffraction
DOS: density of states
VBM: valence band maximum
