# Supplementary material for: Electronic Structure and Surface Chemistry of BaZrS3 Perovskite Powder and Sputtered Thin Film
Source: ACS Appl Mater Interfaces. 2024 Jul 22;16(30):40210–21. doi: 10.1021/acsami.4c06758 (PMC11299144; doi:10.1021/acsami.4c06758)
Supplement: Supplementary file 1 — am4c06758_si_001.pdf [file am4c06758_si_001.pdf]

Supplementary Information

Electronic Structure and Surface Chemistry of $BaZrS_3$ Perovskite Powder and Sputtered Thin Film

Stefania Riva[1], Soham Mukherjee[1,], Sergei M. Butorin[1], Corrado Comparotto[2], Garima Aggarwal[2,†], Evelyn Johannesson[1,4], Mahmoud Abdel-Hafiez [3,1,*], Jonathan Scragg[2], Håkan Rensmo[1,4,*]*

[1]Division of X-ray Photon Science, Department of Physics and Astronomy, Uppsala University, Box 516, Uppsala SE-75120, Sweden

[2]Division of Solar Cell Technology, Department of Materials Science and Engineering, Uppsala University, Uppsala 75237, Sweden

[3]Department of Applied Physics and Astronomy, University of Sharjah, P. O. Box 27272 Sharjah, United Arab Emirates

[4]Wallenberg Initiative Materials Science for Sustainability (WISE), Department of Physics and Astronomy, Uppsala University, Uppsala SE-75120, Sweden

*hakan.rensmo@physics.uu.se

*mahmoud.hafiez@physics.uu.se

*soham.mukherjee@physics.uu.se , soham.chem@gmail.com

1. EXAFS analysis

Table S1. Details of higher NN fits to Zr *K*-XAS data for the $BaZrS_3$ powder and the thin film: bond distances (R), pseudo-Debye-Waller factors (σ^2), coordination numbers (N), amplitude reduction factor (amp), energy correction factors (ΔE_0) and quality of the independent fits.

Scattering atom	Shell no.	*R* (Å)	σ^2 (Å^2)	*N**	*amp*	ΔE_0 (eV)	R-factor
**Powder**							
S	1NN	2.539±0.004	0.0058 ± 0.0005	6	0.96*	-0.7±0.6	0.006
Ba1	2NN	4.077±0.03	0.006 ± 0.004	2			
Ba2		4.212±0.03		2			
Ba3		4.252±0.02		2			
Ba4		4.385±0.03		2			
Zr	3NN	4.835±0.03	0.012 ± 0.003	6			
-S-Zr-		4.874 ±0.03	0.065*	12			
**Thin film**							
S	1NN	2.551±0.005	0.0066 ± 3.1x10^{-4}	6	0.97*	1.3±0.7	0.011
Ba1	2NN	4.14±0.05	0.009*	2			
Ba2		4.22±0.06		2			
Ba3		4.32±0.05		2			
Ba4		4.40±0.05		2			
Zr	3NN	4.89±0.03	0.012*	6			
-S-Zr-		4.96±0.05	0.027 ±0.011	12			

*parameters fixed during EXAFS fits

2. Powder

(a) (b)

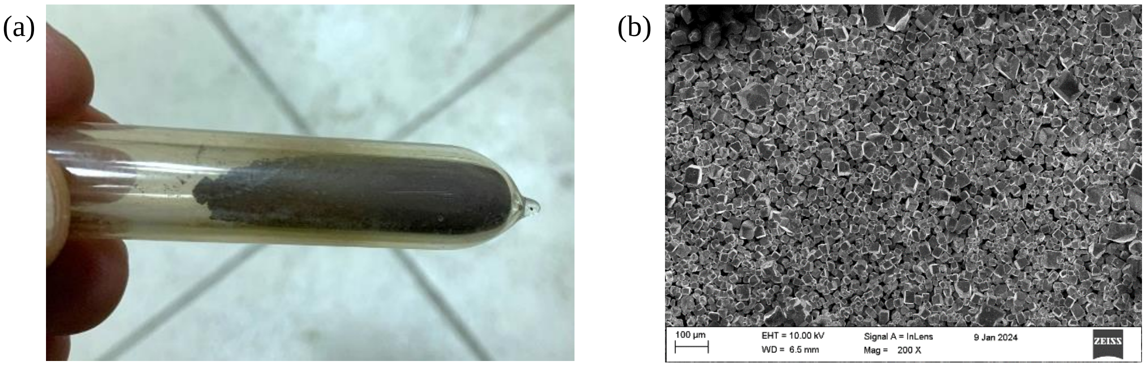

Figure S1: (a) Image showing $BaZrS_3$ powder in sealed ampoule; (b) corresponding SEM image.

3. Cross sections at 9.25 keV

The cross section for XPS accounts for the interaction factor between the core electron and the photon at a specific photon energy. From references, the cross section is tabulated only for sharp photon energy numbers. Therefore, in order to obtain the cross sections for measurements at Ga K_α (9.25 keV), an exponential fit was performed starting from the tabulated cross sections at 6000 eV, 8000 eV and 10000 eV, and the cross section at 9.25 keV was calculated according to the function describing the trend.

For example, to obtain the cross section of Ba $3d_{5/2}$, the fit of the cross section at 6000 eV, 8000 eV and 10000 eV is found to be according to the function: *Cross section* = 69145*exp(-0,0005*$h\nu$), which with hν = 9525.79 eV, the cross section = 676.93.

With the same method, we calculated the cross sections of the shallow core levels Ba 5s, Ba 5p, S 3s, S 3p, Zr 4p, Zr 4d, Zr 4s for the valence band at hν = 9525.79 eV. In this case, the cross section of the levels was calculated by averaging the cross sections of the spin-orbit splitting.

Table S1 reports the tabulated values from Scofield for the core levels Ba 3d, Zr 3p, Zr 3d, S 2p, and Table S2 for the shallow levels Ba 5s, Ba 5p, S 3s, S 3p, Zr 4p, Zr 4d, Zr 4s.

Table S2: Cross section values for the selected core levels of interest for $BaZrS_3$ at the tabulated photon energies of 1500 eV, 6000 eV, 8000 eV, 10000 eV. The cross sections at 9252.79 eV were calculated from the exponential fit.

	**hν (eV)**				
**Core level**	*1500*	*6000*	*8000*	*10000*	*9252.79*
**Ba $3d_{5/2}$**	343980	4464.9	1598.7	702.48	676.93
**Zr $3p_{3/2}$**	68778	2308.4	1006.3	515.54	525.80
**S $2p_{3/2}$**	14700	133.36	46.585	20.292	82.46
**O 1s**	38989	682.05	281.04	140.04	175.40
**Zr $3d_{5/2}$**	55251	418.95	135.61	55.236	21.20

Table S3: Cross section values for the selected core levels of interest of the valence band for $BaZrS_3$ at the tabulated photon energies of 6000 eV, 8000 eV, 10000 eV. The cross sections at 9252.79 eV were calculated from the exponential fit.

**Core level**		**hν (eV)**				
**BARIUM**	*1500*	*6000*	*8000*	*10000*	*9252.79*	*9252.79 avg*
**Ba 6s**	275	20.66	11.543	7.2555	6.06	6.06
**Ba $5p_{1/2}$**	2712.5	156.54	77.63	43.913	64.21	92.22
**Ba $5p_{3/2}$**	5362.5	273.94	132.44	73.411	120.25	
**ZIRCONIUM**						
**Zr 5s**	465.89	30.92	16.563	10.048	10.18	10.18
**Zr $4p_{1/2}$**	4781.1	170.72	75.938	39.534	36.85	53.07
**Zr $4p_{3/2}$**	9221.7	308.3	135.11	69.482	69.29	
**Zr $4d_{3/2}$**	462.46	4.0068	1.3212	0.54557	0.75	0.91
**Zr $4d_{5/2}$**	667.26	5.6012	1.8337	0.75254	1.07	
**SULFUR**						
**S 3s**	1959	67.146	30.915	16.651	33.0	33.0
**S $3p_{1/2}$**	348.01	3.462	1.2249	0.53854	0.53	0.78
**S $3p_{3/2}$**	680.03	6.639	2.3374	1.0233	1.04	

4. DOS and experimental valence band at Al K_{α}

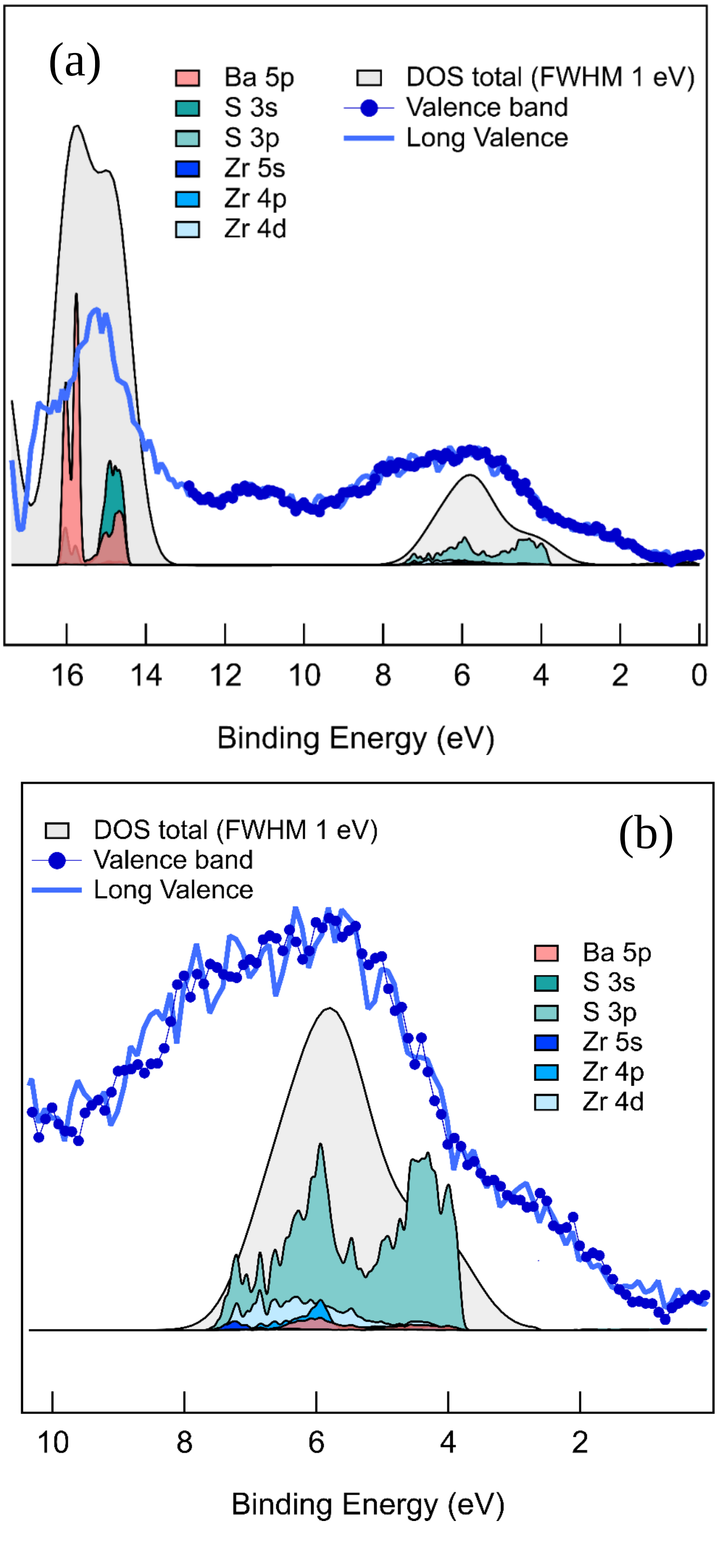

Figure S2: Calculated density of states, weighted by the cross section at 1500 eV, compared with the experimental valence band measurement at 1487 eV for the powder: (a) over a binding energy range up to 18 eV; (b) focus on the valence band region (0 to 10 eV). Energy calibration was performed vs Fermi level of the thin film measured at Ga K_{α} (namely, S $2p_{3/2}$ at 161.15 eV).
